# Complete Genomic Sequence of Canine Distemper Virus from an Ethiopian Wolf

**DOI:** 10.1128/genomeA.00621-17

**Published:** 2017-07-20

**Authors:** Denise A. Marston, Jemma Watson, Emma L. Wise, Richard J. Ellis, Eric Bedin, Girma Ayalew, Muktar Abute, Xavier de Lamballerie, Anthony R. Fooks, Claudio Sillero-Zubiri, Ashley C. Banyard

**Affiliations:** a Animal and Plant Health Agency (APHA), Surrey, United Kingdom; b UMR “Émergence des Pathologies Virales” (EPV) Aix-Marseille University, IRD 190, INSERM 1207, EHESP, IHU Méditerranée Infection, Marseille, France; c Wildlife Conservation Research Unit, The Recanati-Kaplan Centre, Zoology, University of Oxford, Oxford, United Kingdom; d Ethiopian Wolf Conservation Programme, Dinsho, Ethiopia; e Ethiopian Wildlife Conservation Authority, Addis Ababa, Ethiopia; f Institute of Infection and Global Health, University of Liverpool, Liverpool, United Kingdom

## Abstract

Canine distemper virus (CDV) has been implicated in population declines of wildlife, including many threatened species. Here we present the full genome of CDV from an Ethiopian wolf, *Canis simensis*, the world’s rarest and most endangered canid.

## GENOME ANNOUNCEMENT

Canine distemper virus (CDV), a member of the *Morbillivirus* genus, has a negative-strand nonsegmented RNA genome. CDV has recently been implicated in population declines of numerous wildlife species, including lions (*Panthera. leo*) (1), black-backed jackals (*Canis mesomelas*) (2), fennec foxes (*Vulpes zerda*) (3), spotted hyenas (*Crocuta crocuta*) (4), and rhesus monkeys (*Macaca mulatta*) (5), and aquatic species, including Lake Baikal seals (*Phoca siberica*) and Caspian seals (*Phoca caspia*) (6). Significantly, CDV has had an impact on several threatened species, including the world’s most endangered felid, the Iberian lynx (*Lynx pardinus*) (7), and the Amur tiger (*Panthera tigris. altaica*) (8), the Santa Catalina Island fox (*Urocyon littoralis catalinae*) (9), and the Ethiopian wolf (*Canis simensis*) (10). Historically, different strains of CDV were grouped geographically (11); however, an increased interest in CDV and the potential for translocation of infected animals has revealed that CDV phylogenies are complex, much like those of the related measles virus, presenting a multilineage global distribution (12). Here we describe full-genome sequencing of a CDV derived from an infected subadult male Ethiopian wolf from the Sodota pack, found dead during an outbreak in the Web Valley, Bale Mountains, southeastern Ethiopia, in September 2015 (CDV06) (10).

RNA from spleen stored in glycerol was prepared for next-generation sequencing on the MiSeq platform. Briefly, TRIzol-extracted viral RNA was depleted of host genomic DNA and rRNA as described previously (13). Double-stranded (ds) cDNA was synthesized from 50 ng RNA, using a random cDNA synthesis system (Roche), according to the manufacturers’ instructions. The ds cDNA was purified using Ampure XP magnetic beads (Beckman Coulter, Inc.), and 1 ng was used for the Nextera XT DNA sample preparation kit (Illumina). A sequencing library was prepared and sequenced on an Illumina MiSeq with 2- × 150-bp paired-end reads. The total reads (5,354,417) were mapped to a reference sequence (GenBank accession number JN896331) using BWA (v 0.7.5a-r405) (14), and visualized in Tablet (15). A modified SAMtools/vcfutils (16) script was used to generate an intermediate consensus sequence in which any indels relative to the original reference sequence were appropriately called. The intermediate consensus was used as the reference for subsequent iteration of mapping and consensus calling. The total number of assembled viral reads was 387,043 (7.23% of the total reads). Despite the low proportion of viral sequence detected within the total data set, coverage of the entire genome was obtained (average read depth of 1,798).

The genetic organization of the Ethiopian wolf CDV genome was consistent with those of other CDV genomes, with a complete genome size of 15,690 nucleotides. The genome has closest homology (99%) to a sequence from China (GenBank accession number JN896331) clustering with Asia-1 sequences rather than African CDV sequences, including those from neighboring Tanzania.

### Accession number(s).

The complete genomic sequence of CDV06 has been deposited in GenBank under accession number MF041963.
